# GEOGRAPHIC DISPARITIES IN LIFE EXPECTANCY AND MORTALITY IN THE U.S.

**DOI:** 10.1093/geroni/igz038.1597

**Published:** 2019-11-08

**Authors:** Julia Kravchenko, H Kim Lyerly

**Affiliations:** 1 Duke University School of Medicine, Durham, North Carolina, United States; 2 Duke University Medical Center, Durham, North Carolina, United States

## Abstract

Although the US has one of the highest per-capita health expenditures in the world, it noticeably lags behind a number of other industrialized countries in terms of life expectancy (LE). These disparities remain unexplained by individual demographic, socioeconomic, and healthcare factors. Analysis of death certificates for 1999-2016 revealed that diseases contributed most to LE variability are myocardial infarction (explained 12.9% of the difference in mortality), heart failure (10.6%), stroke (8.2%), lung cancer (7.5%) and COPD (7.2%). Analysis of histories of diseased patients in Medicare records showed that septicemia (15.7%), low weight (13.8%), renal disease (13.3%), disorders of electrolyte and fluid balance (9.0%) and heart failure (7.3%) contributed most to the disparities. Diseases that substantially contribute to disparities in LE in the US include both common and less-often-discussed diseases. Future studies of variations in treatment patterns, access-to and quality-of medical care for these diseases could provide important insight in observed patterns.

